# Novel Variants and Clinical Heterogeneity in Pediatric Calcium Metabolism Disorders Identified Through High-Yield Tiered Genetic Testing in a Taiwanese Cohort

**DOI:** 10.3390/medicina61101861

**Published:** 2025-10-16

**Authors:** Ting-Yu Kang, Yen-Yin Chou, Yu-Ming Chang, Yu-Wen Pan, Meng-Che Tsai

**Affiliations:** 1 Department of Pediatrics, National Cheng Kung University Hospital, College of Medicine, National Cheng Kung University, Tainan 704302, Taiwan; ericakang12@gmail.com (T.-Y.K.); yenyin@mail.ncku.edu.tw (Y.-Y.C.); puddingyd@gmail.com (Y.-M.C.); 2 Department of Genomic Medicine, National Cheng Kung University Hospital, College of Medicine, National Cheng Kung University, Tainan 704302, Taiwan

**Keywords:** calcium metabolism, hypoparathyroidism, pseudohypoparathyroidism, CASR, GNAS, genetic diagnosis

## Abstract

*Background and Objectives*: Inherited disorders of calcium metabolism are rare pediatric conditions with diverse manifestations, including seizures, growth impairment, and renal or skeletal complications. Precise molecular diagnosis is crucial for effective management and informed genetic counseling. This study aimed to develop a systematic diagnostic approach, broaden the mutational spectrum, and characterize initial clinical features. *Material and Methods*: We retrospectively analyzed 13 pediatric cases at a tertiary center in southern Taiwan (2020–2025). Clinical, biochemical, and imaging data were reviewed. Genetic testing followed a tiered strategy to identify copy number variations and single-nucleotide variants. Variants were classified according to the ACMG/AMP guidelines and assessed by in silico tools. *Results*: The pediatric cohort (8 males, 5 females) had a median diagnostic age of 2 years and a mean follow-up of 7.7 years. Hypoparathyroidism was most common (*n* = 7), followed by PTH resistance (*n* = 3), hyperparathyroidism (*n* = 1), calcipenic rickets (*n* = 1), and syndromic hypercalcemia (*n* = 1). Genetic diagnoses were established in 12 children and one parent, involving *CASR*, *GNAS*, *PRKAR1A*, *CYP27B1*, and *KMT2D*. Two novel variants were identified (*CASR* p.Val836Ile and *GNAS* c.719-30A>T). Phenotypic heterogeneity included incomplete penetrance in autosomal dominant hypocalcemia and variable multisystem involvement in syndromic cases. *Conclusions*: A stepwise genetic testing strategy achieved a high diagnostic yield in pediatric calcium metabolism disorders. The discovery of novel and population-specific variants expands the mutational spectrum, supporting precision medicine in pediatric endocrinology.

## 1. Introduction

Calcium is an essential mineral involved in numerous biological processes, including skeletal mineralization, neuromuscular excitability, blood coagulation, and intracellular signaling. Homeostasis is tightly regulated by the parathyroid glands, intestine, kidneys, and bones to maintain serum calcium within a narrow physiological range [1]. In pediatric populations, rapid growth and a high demand for skeletal mineralization necessitate increased parathyroid hormone (PTH) and 1,25-dihydroxyvitamin D levels, often preserving near-normal serum calcium and phosphate despite underlying pathology. As a result, skeletal manifestations may be the earliest or only signs of disease, with biochemical abnormalities remaining subtle or absent [1,2,3]. In contrast, adults typically maintain neutral calcium balance with lower skeletal mineral requirements. Acquired causes, most notably postsurgical hypoparathyroidism, predominate and often present with overt biochemical disturbances such as hypocalcemia and hyperphosphatemia [4,5,6]. These physiological and etiological distinctions emphasize the need for age-specific diagnostic strategies and heightened clinical awareness in children.

Inherited disorders of calcium metabolism encompass a heterogeneous spectrum of diseases, many of which present during childhood and are associated with significant morbidity. These disorders can be broadly categorized into defects of calcium homeostasis, such as hypoparathyroidism, PTH resistance, and hyperparathyroidism. Additionally, abnormalities in vitamin D metabolism or action, caused by mutations in *CYP27B1, CYP2R1*, or *VDR*, contribute to the spectrum of hereditary calcipenic rickets [7,8,9]. The clinical manifestations of these disorders are diverse, ranging from seizures due to hypocalcemia, growth retardation, skeletal deformities, to renal complications, and multisystem involvement in syndromic conditions such as DiGeorge syndrome and Williams syndrome. Management is particularly challenging, as inadequate treatment may result in recurrent seizures or bone fragility, whereas overtreatment predisposes to hypercalciuria, nephrocalcinosis, and renal impairment [10,11,12].

Molecular genetic studies have identified pathogenic variants in a wide range of genes, including *CASR*, *GNAS*, *PRKAR1A*, *PTH*, *SOX3*, *GCMB*, and *CYP27B1* [13,14,15]. However, considerable variability exists in disease severity, onset, and systemic involvement. While some mutations lead to isolated endocrine dysfunction, others contribute to syndromic presentations with developmental, cardiac, or immunological abnormalities. Because of this genetic and phenotypic heterogeneity, the selection of appropriate genetic tests is complex, highlighting the need for a structured, phenotype-led diagnostic algorithm. Recent guidance supports the use of next-generation sequencing panels combined with multiplex ligation-dependent probe amplification (MLPA) to capture both sequence and copy-number variants in key genes implicated in pediatric calcium and phosphate disorders [16,17].

Accurate molecular diagnosis also provides opportunities to link genotypes with clinical features and to tailor management strategies. For instance, the American Academy of Pediatrics recommends routine screening of serum calcium and PTH in children with 22q11.2 deletion syndrome, with molecular confirmation guiding management and surveillance for associated comorbidities [18]. In CYP27B1-related rickets, genotype–phenotype correlations inform disease severity and therapeutic requirements; for instance, the c.195+2T>G splice-site variant has been linked to more severe phenotypes and higher calcitriol needs [19,20]. Together, these findings support our emphasis on integrating molecular data into longitudinal care.

In this study, we conducted a comprehensive genetic survey of pediatric patients with suspected inherited disorders of calcium metabolism at a single tertiary center in Taiwan. The aims were to establish a comprehensive and cost-effective diagnostic strategy, to expand the mutational spectrum by detecting novel pathogenic variants, and to characterize the initial clinical presentations and potential underlying mechanisms in these pediatric patients.

## 2. Material and Methods

### 2.1. Study Population

This retrospective study was conducted at National Cheng Kung University Hospital (NCKUH) from 2020 to 2025. Patients were enrolled if they were clinically suspected of having a disorder of calcium metabolism, presented before the age of 18 years, and had been diagnosed at our hospital within the past 30 years. For those without a prior confirmed molecular diagnosis, genetic testing was performed.

Clinical diagnostic classifications were established according to standardized biochemical criteria: hypoparathyroidism was defined as decreased calcium level accompanied by low or inappropriately normal iPTH; PTH resistance as decreased calcium level with elevated iPTH; calcitropic rickets as hypocalcemia with/without hypophosphatemia, elevated alkaline phosphatase, and clinical evidence of bone softening and weakening, such as genu varum; primary hyperparathyroidism as elevated calcium level with non-suppressed iPTH; and syndromic hypercalcemia as elevated calcium with no evidence of increased iPTH or vitamin D level. Patients with acquired or secondary causes of calcium and/or phosphate imbalance, including nutritional rickets, chronic kidney disease, tumor lysis syndrome, or postsurgical hypoparathyroidism, were excluded from the study. A total of 13 pediatric patients were included.

Clinical information, including demographic data, birth and family history, age at symptom onset and diagnosis, initial presentation, physical findings, associated anomalies, laboratory results, imaging findings, treatments, and complications, was collected from both electronic and paper-based medical records.

This study was approved by the Institutional Review Board of National Cheng Kung University Hospital. The patient and the legal guardian signed informed consents, as required for patients aged 7 to 18 years old, and by the legal guardian for patients under 7 years old.

### 2.2. Genetic Testing

**Figure 1:**
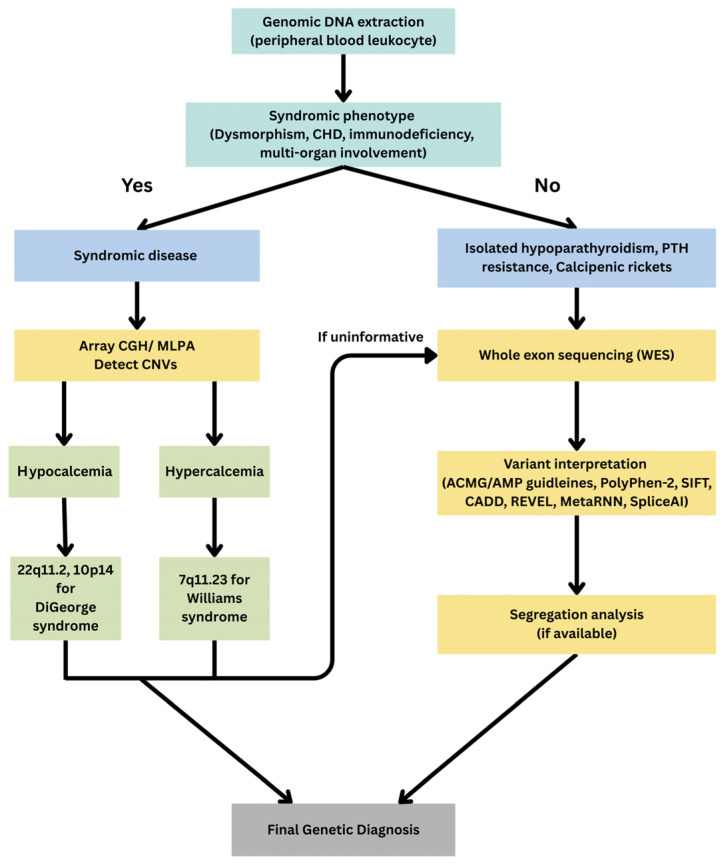
Diagnostic algorithm.

Genomic DNA was extracted from peripheral blood leukocytes using standardized protocols. Genetic analysis followed a tiered approach tailored to the patient’s clinical presentation. First, patients with syndromic phenotypes—such as dysmorphic features, congenital heart defects, or associated congenital anomaly—underwent copy number variation testing via array comparative genomic hybridization (array CGH) or multiplex ligation-dependent probe amplification (MLPA) to identify pathogenic deletions or duplications. If first-tier testing was uninformative, whole-exome sequencing (WES) was performed to detect single-nucleotide variants and small insertions or deletions. Candidate variants were confirmed by bidirectional Sanger sequencing. Variant interpretation adhered to the American College of Medical Genetics and Genomics/Association for Molecular Pathology (ACMG/AMP) guidelines, incorporating multiple in silico prediction algorithms, including PolyPhen-2 (Polymorphism Phenotyping v2; http://genetics.bwh.harvard.edu/pph2/, accessed on 28 August 2025), SIFT (Sorting Intolerant From Tolerant; https://sift.bii.a-star.edu.sg/, accessed on 28 August 2025), CADD (Combined Annotation Dependent Depletion; https://cadd.gs.washington.edu/, accessed on 28 August 2025), REVEL (Rare Exome Variant Ensemble Learner; https://sites.google.com/site/revelgenomics/, accessed on 28 August 2025), MetaRNN (https://github.com/Chang-Li2019/MetaRNN, accessed on 28 August 2025), HSF (Human Splicing Finder; https://hsf.genomnis.com/mutation/analysis, accessed on 28 August 2025), and SpliceAI (https://spliceailookup.broadinstitute.org/, accessed on 28 August 2025). Segregation analysis was conducted when DNA samples from family members were available. The diagnostic algorithm is depicted in Figure 1.

## 3. Results

From 2020 to 2025, we consecutively enrolled children evaluated for suspected inherited disorders of calcium metabolism at a single tertiary center. This study comprised 13 pediatric patients, including 8 males and 5 females, with a median age at diagnosis of 2 years (range: 1 month to 36 years), and a mean follow-up duration of 7.7 ± 6.5 years (range: 0.5–20 years). One adult patient, the father of patient 3, was asymptomatic but found to harbor the same CASR variant; he was tested for familial segregation and was not included in pediatric analyses.

We molecularly diagnosed mutations in *CASR*, *KMT2D*, *GNAS*, *PRKAR1A*, and *CYP27B1*, corresponding to cases with clinical diagnoses of isolated hypoparathyroidism/primary hyperparathyroidism, syndromic hypoparathyroidism, pseudohypoparathyroidism, acrodysostosis, and calcipenic rickets.

**Figure 2:**
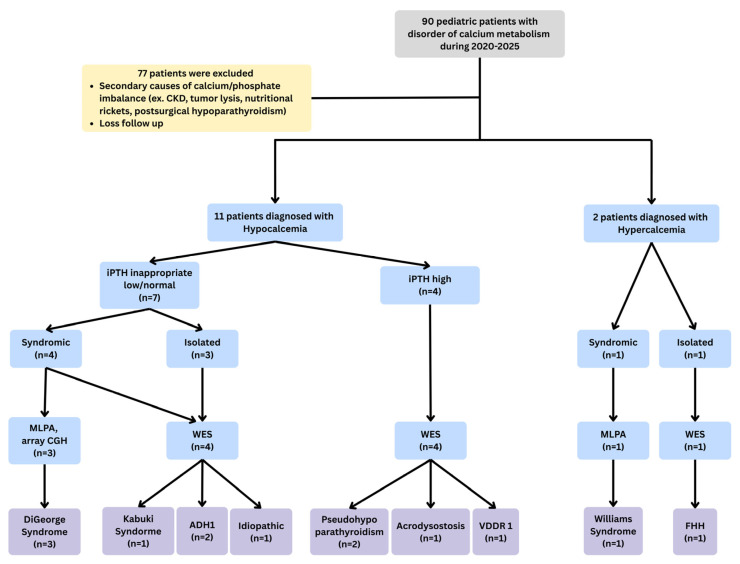
Patients’ final molecular diagnosis. Definitive genetic diagnosis was established in 12 of 13 cases, yielding a 92.3% molecular diagnostic rate.

The patient characteristics were summarized in Table 1. The final molecular diagnosis was presented in Figure 2.

### 3.1. Hypoparathyroidism

Among the 7 patients diagnosed with hypoparathyroidism, 3 were classified as isolated hypoparathyroidism and 4 had syndromic forms (including DiGeorge syndrome and Kabuki syndrome), with a median age at diagnosis of 1 month. Six patients presented with seizures as the initial symptom, whereas one was asymptomatic and diagnosed because of his affected son.

Serum calcium, phosphate, and iPTH profiles reflected typical biochemical patterns of hypoparathyroidism and ADH1. The mean serum calcium level at diagnosis was 6.5 ± 0.9 mg/dL (range 5.3–7.6 mg/dL), the mean serum phosphate level was 8.1 ± 1.4 mg/dL (range 4.5–10.2 mg/dL), and the mean intact parathyroid hormone (iPTH) level was 12.1 ± 4.0 pg/mL (range 7.9–20 pg/mL), which were inappropriately low or low-normal. These biochemical features are characteristic of impaired PTH secretion or action. Other presentations included calcifications in the basal ganglia or other deep brain nuclei observed on brain ultrasonography or MRI.

#### 3.1.1. Isolated Hypoparathyroidism

We identified 2 *CASR* mutations in 3 cases with isolated hypoparathyroidism. One patient (P2) had a previously reported pathogenic variant, p.Val836Leu, which was associated with hypercalciuria, leading to the molecular diagnosis of ADH1. The remaining two patients—a father-son pair (P3)—harbored a novel *CASR* missense variant, p.Val836Ile. Multiple in silico predictors, including CADD, REVEL, PolyPhen-2, and MetaRNN, predicted this variant to be pathogenic. The son presented with hypocalcemic symptoms at infancy, while the father remained asymptomatic, suggesting variable expressivity and incomplete penetrance. The last patient (P1) had no identifiable genetic variant and was classified as idiopathic hypoparathyroidism.

The adult father exhibited a borderline ionized calcium level of 1.03 mmol/L, alongside a phosphate level of 3.3 mg/dL and a relatively elevated iPTH of 27.6 pg/mL. This suggests a compensatory hormonal response despite mild hypocalcemia, and highlights incomplete penetrance in autosomal dominant hypocalcemia type 1 (ADH1) due to a *CASR* mutation.

#### 3.1.2. Syndromic Hypoparathyroidism

Four patients were diagnosed with syndromic hypoparathyroidism and exhibited a broad range of systemic manifestations beyond calcium-phosphate abnormalities. These patients presented in infancy or early childhood with hypocalcemic seizures, and all had dysmorphic features and associated anomalies.

Three patients were genetically confirmed to have DiGeorge syndrome, including two with 22q11.2 microdeletion (type 1) and one with a 10p14 deletion (type 2), either by MLPA or array CGH. Patients (P6, P7) with 22q11.2 deletions exhibited more prominent hypocalcemic seizures in the neonatal period, T-cell immunodeficiency, as evidenced by abnormal newborn screening TREC results and decreased CD3+ counts. Both had facial dysmorphism; one had an undescended testicle with a patent foramen ovale, and the other had an atrial septal defect and patent ductus arteriosus, consistent with the classic multisystem involvement of 22q11.2DS. In contrast, the patient (P6) with a 10p14 deletion (likely HDR-related) showed milder hypocalcemia and immunodeficiency but had renal dysplastic changes with atrophy and a ventricular septal defect, consistent with features more commonly associated with type 2 DiGeorge phenotype. These observations highlight the phenotypic distinctions between different genetic subtypes of DiGeorge syndrome.

One patient (P7) was diagnosed with Kabuki syndrome due to a pathogenic variant at the *KMT2D* gene (c.5993A > G, p.Tyr1998Cys), which was previously reported [21]. In addition to seizures and hypocalcemia, this patient had growth hormone deficiency, bilateral hearing loss, short stature, hirsutism, and renal abnormalities, representing several atypical manifestations of Kabuki syndrome. These findings suggest the possibility of additional coexisting problems that warrant further evaluation.

### 3.2. PTH Resistance

Three patients exhibited features consistent with parathyroid hormone (PTH) resistance. Two were diagnosed with pseudohypoparathyroidism due to *GNAS* mutations and one with acrodysostosis caused by a *PRKAR1A* variant. The median age at diagnosis was 9 years (range: 6–14 years), which may reflect the more subtle and progressive nature of the phenotype. Biochemically, the serum calcium level was within the lower range of normal. The mean calcium level at diagnosis was 8.3 ± 0.2 mg/dL (range 8.1–8.5 mg/dL). The mean phosphate level was 5.9 ± 0.7 mg/dL (range 5.4–6.8 mg/dL). The iPTH levels were markedly elevated, with a mean of 251.7 ± 76.6 pg/mL (range 177.7–330 pg/mL), reflecting classical biochemical hallmarks of PTH resistance.

#### 3.2.1. Pseudohypoparathyroidism

Two patients were diagnosed with pseudohypoparathyroidism and carried heterozygous mutations in the *GNAS* gene. One (P10) had a frameshift duplication (c.74dup, p.Ile26AspfrTer28), while the other (P9) carried a rare and novel splice site mutation (c.719-30A>T). This rare variant is absent from gnomAD, was found to be de novo, and is clinically consistent with the diagnosis of pseudohypoparathyroidism. SpliceAI predicted a score of 0.55, indicating a potential splice effect, and Human Splicing Finder predicted that the A>T change disrupts the natural (wild-type) branch point motif upstream of the 3′ splice site. Such disruption may impair normal spliceosome recognition, potentially resulting in exon skipping, intron retention, or activation of a cryptic acceptor site, thereby altering the splicing process.

Both patients exhibited typical features of PTH resistance, including biochemical hypocalcemia with markedly elevated iPTH levels (177.7 and 330 pg/mL, respectively), as well as clinical findings of short stature, brachydactyly, round face, and skeletal anomalies. In addition, both patients demonstrated multi-hormone resistance: each had hypothyroidism requiring thyroxin replacement and either irregular menstruation or hypogonadism with primary amenorrhea. Neither patient showed renal complications during follow-up. The novel splice site variant expands the mutational spectrum of *GNAS*-related disorders and highlights the phenotypic consistency among individuals with molecularly confirmed pseudohypoparathyroidism.

#### 3.2.2. Acrodysostosis

One patient (P8) was diagnosed with acrodysostosis and carried a heterozygous pathogenic variant in the *PRKAR1A* gene (c.1004 G>T). This patient also presented with biochemical features of PTH resistance, including hypocalcemia and significantly elevated iPTH (247.4 pg/mL). Clinically, the patient exhibited short stature, brachydactyly, cone-shaped epiphyses of fingers, and hypothyroidism, overlapping with features seen in pseudohypoparathyroidism but consistent with acrodysostosis due to the distinct genetic etiology.

### 3.3. Vitamin D-Dependent Rickets Type 1A

One patient (P11) was diagnosed with vitamin D-dependent rickets type 1A and carried compound heterozygous mutations in the *CYP27B1* gene, including a missense variant (c.1166 G>A, p.Arg289His) and a frameshift variant (c.1319_1325dup, p.Phe443ProfsTer24). The patient presented at 1 year of age with clinical features of rickets, including genu varum and growth delay. Laboratory findings revealed hypocalcemia (7.3 mg/dL), hypophosphatemia (2.4 mg/dL), and markedly elevated iPTH (321.7 pg/mL), consistent with secondary hyperparathyroidism. During treatment with calcium carbonate and calcitriol, despite the presence of hypercalciuria, renal ultrasound revealed bilateral renal parenchymal changes without evidence of nephrocalcinosis. The patient’s clinical symptoms and growth parameters gradually improved during follow-up.

### 3.4. Familial Hypocalciuric Hypercalcemia

One patient (P12) was diagnosed with primary hyperparathyroidism and presented at 7 years of age with intermittent arthralgia. Biochemical evaluation revealed hypercalcemia (11.7 mg/dL), hypophosphatemia (3.9 mg/dL), and an elevated iPTH level of 76.2 pg/mL, consistent with excessive PTH secretion. A parathyroid scan showed no evidence of parathyroid adenoma. Genetic testing identified a heterozygous missense variant in the *CASR* gene (c.1661T>A, p.Ile554Asn), which was classified as pathogenic according to ACMG criteria. This variant has also been previously reported in a Taiwanese patient with functional testing performed [22]. The clinical and molecular findings were consistent with familial hypocalciuric hypercalcemia potentially caused by a loss-of-function *CASR* mutation. He was closely monitored without immediate pharmacologic intervention, and no renal or skeletal complications were noted during follow-up.

### 3.5. Williams Syndrome

One pediatric patient (P13) was diagnosed with Williams syndrome due to a heterozygous 7q11.23 microdeletion. The child presented at 2 years of age with failure to thrive, neurodevelopmental delay, hypotonia, hearing impairment, hypospadias, and distinctive dysmorphic features, including frontal bossing, hypertelorism, and irregularly spaced teeth. Laboratory evaluation revealed hypercalcemia (16.0 mg/dL), hypophosphatemia (3.9 mg/dL), and an undetectable iPTH (<1.2 pg/mL), consistent with hypercalcemia secondary to Williams syndrome. Management consisted of dietary control without pharmacologic intervention.

## 4. Discussion

In our small, single tertiary center cohort of pediatric patients with disorders of calcium metabolism, the most common condition was hypoparathyroidism (*n* = 7), followed by PTH resistance (*n* = 3), hyperparathyroidism (*n* = 1), calcipenic rickets (*n* = 1), and syndromic hypercalcemia (*n* = 1). After excluding cases with secondary causes of calcium and phosphate disequilibrium, 12 of the 13 pediatric patients were found to have a known genetic etiology, underscoring that most congenital pediatric cases with calcium and phosphate disorders are genetic in origin. We also identified pathogenic or likely pathogenic variants in the majority of patients, including two novel mutations.

The majority of our isolated hypoparathyroidism cases harbored heterozygous *CASR* mutations, including V836L and the novel V836I variant. While the previously reported p.Val836Leu (V836L) is known to be pathogenic, our patient’s isoleucine substitution at the same codon is predicted by multiple in silico tools to be pathogenic as well. The calcium-sensing receptor (CASR) is a class C G protein-coupled receptor composed of multiple structural domains, including an N-terminal signal peptide, an extracellular domain with calcium-binding sites, a cysteine-rich linker, seven transmembrane helices, and a C-terminal intracellular tail for downstream signaling [23,24]. CASR senses extracellular calcium to regulate PTH secretion and renal calcium handling. Gain-of-function mutations in the TM6–TM7 transmembrane region of *CASR* drive ADH1 by increasing receptor sensitivity to calcium, lowering the PTH suppression threshold, and causing hypocalcemia with low PTH and hypercalciuria. Variants such as p.Val836Leu disrupt the transmembrane helix 6/extracellular loop 3/transmembrane helix 7 (TM6/ECL3/TM7) “molecular switch.” This disruption leads to constitutive or left-shifted activation. In silico predictions, together with functional studies, confirm that TM6/TM7 variants lower the EC50 for Ca^2+^-stimulated signaling [25]. Given the structural similarity between isoleucine and leucine as hydrophobic residues, the novel V836I variant is also likely to be pathogenic. Management involves cautious calcium and calcitriol titration, sometimes thiazides, and investigational calcilytics (e.g., NPSP795/encaleret) to raise PTH and serum calcium while reducing urinary calcium loss [26,27,28].

In our case, the variant also demonstrated incomplete penetrance, as the asymptomatic father carried the same mutation and exhibited only borderline hypocalcemia, accompanied by a compensatory elevation of iPTH. This aligns with previous reports indicating variable expressivity and intrafamilial heterogeneity of *CASR*-related disorders [10,29]. Within the same family, some members with identical *CASR* mutations present only with mild neuromuscular symptoms, while others develop brain calcifications or seizures. Emphasizing that not all carriers in the same family are symptomatic and highlighting the need for family screening and longitudinal surveillance of carriers [29,30].

The genetic spectrum of isolated hypoparathyroidism demonstrates substantial regional and ethnic variation. In Western cohorts, *CASR* gain-of-function mutations are among the most frequently reported causes of autosomal dominant hypocalcemia, whereas *GCM2* mutations are relatively rare but have been described in Latin American and South Asian families, sometimes linked to founder effects [31,32,33,34]. In East and Southeast Asia, *CASR* mutations are more often identified in both familial and sporadic cases, while *GCM2* mutations are extremely uncommon. Most congenital hypoparathyroidism cases reported from Asia are syndromic, particularly those associated with 22q11.2 deletions [35,36,37]. Other genes such as *PTH*, *GNA11*, and *SOX3* are infrequently reported worldwide, without clear evidence of geographic clustering.

Among the four cases with syndromic hypoparathyroidism, three had DiGeorge syndrome, all of whom initially presented with neonatal seizures secondary to severe hypocalcemia. Our cases align with studies demonstrating that 22q11.2 deletions typically cause early-onset hypocalcemia, conotruncal heart defects, and T-cell deficiency, whereas 10p14/HDR deletions are characterized by renal dysplasia and sensorineural hearing loss when present, and usually spare the immune system unless the deletion extends proximally [38,39,40]. Our results also parallel prior Asian cohort studies, including the Korean series reported by Kim et al., which identified 22q11.2 deletion as the leading cause of pediatric hypoparathyroidism and emphasized the risk of renal complications during long-term management [14].

Mechanistically, hypoparathyroidism in these syndromes results from impaired development of the third pharyngeal-pouch. In DiGeorge syndrome type 1 (22q11.2 deletion), *TBX1*—a dosage-sensitive regulator of pharyngeal arch/pouch morphogenesis—is essential for parathyroid and thymic primordia [41,42]; haploinsufficiency disrupts pharyngeal segmentation and *FGF8/FGF10* signaling, reducing thymic epithelial-cell proliferation, migration, and the number of *GCM2*-positive parathyroid progenitors. *GCM2* is essential for the differentiation and survival of parathyroid precursors [43,44,45,46]. *CRKL*, an adaptor in FGF signaling encoded in the central 22q11.2 interval, cooperates with *TBX1*, and loss of *CRKL* reduces FGF8 responses [47,48]. Hypoparathyroidism occurs in approximately 17–60% of 22q11.2DS cases, typically in the neonatal period, with potential recurrence later in life [49,50]. Phenotypic variability partly reflects which 22q11.2 interval is deleted, proximal A–B (including *TBX1*, *DGCR8*) vs. central B–D (including *CRKL*) vs. distal D–E (including *MAPK1*), which differentially impact cardiac, immune, and neurodevelopmental features [51,52]. *GATA3* haploinsufficiency at 10p14 causes hypoparathyroidism with renal/otic anomalies and generally spares T-cell development, because *GATA3* expression in early T-cell progenitors is regulated by a monoallelic-to-biallelic transcriptional switch that compensates for the loss of one allele and maintains sufficient protein levels for normal T-cell maturation [53,54,55,56].

A previously reported patient with a *KMT2D* variant (c.5993A>G, p.Tyr1998Cys) demonstrated an expanded Kabuki syndrome phenotype characterized by endocrine and renal involvement rather than classic craniofacial features. The clinical presentation included progressive ataxia, developmental regression, myoclonus, bilateral sensorineural hearing loss, growth hormone deficiency, and primary hypoparathyroidism. Whole-exome sequencing identified the *KMT2D* variant, along with compound heterozygous VPS13D variants, which explains the combined Kabuki syndrome and SCAR4 phenotype [21]. Recent reports have described patients presenting with unusual clinical features, including hypocalcemia and hypoparathyroidism [57,58]. This case also supports evidence that certain missense *KMT2D* variants can manifest as atypical or endocrine-predominant phenotypes with neurologic involvement.

The second most common condition was PTH resistance. Two cases harbored heterozygous *GNAS* mutation, including a novel splice site variant (c.719-30A>T), compatible with pseudohypoparathyroidism, and one had a heterozygous *PRKAR1A* mutation, compatible with acrodysostosis. Compared with hypoparathyroidism cases presenting as neonatal seizures, cases with PTH resistance often initially present with short stature.

Pseudohypoparathyroidism type 1A (PHP1A) is caused by maternally inherited mutations or epigenetic alterations in the *GNAS* gene, which encodes the stimulatory G protein α-subunit (Gsα). Due to tissue-specific imprinting, only the maternal allele is expressed in hormone-sensitive tissues such as the renal proximal tubule, thyroid, gonads, and pituitary. Loss of function of the maternal allele leads to resistance to hormones that signal via Gsα, particularly parathyroid hormone (PTH) [59,60]. Early in life, residual paternal Gsα expression may delay biochemical resistance, but as imprinting becomes fully established, PTH resistance emerges clinically. AHO features such as short stature and brachydactyly appear early due to disrupted PTHrP signaling in growth plates, which accelerates chondrocyte maturation and epiphyseal closure [61,62,63]. Growth deceleration often begins in childhood, frequently before hypocalcemia develops, making skeletal features an early diagnostic clue. In addition to classic coding mutations, pathogenic mechanisms include methylation defects and deletions of imprinting control regions, which may further impair imprinting regulation and downstream endocrine signaling. These genetic and epigenetic complexities contribute to the heterogeneous and age-dependent phenotype of PHP1A [64,65,66].

Acrodysostosis presents with more profound skeletal abnormalities than pseudohypoparathyroidism, including generalized brachydactyly, advanced bone age, and craniofacial dysostosis. This is because the mutations in acrodysostosis (*PRKAR1A* or *PDE4D*) disrupt cAMP signaling downstream of the *GNAS*-encoded Gsα protein, leading to a broader and more severe impairment of the cAMP/protein kinase A (PKA) pathway in chondrocytes and osteoblasts, which are critical for endochondral bone formation and skeletal patterning [67,68]. In particular, *PRKAR1A* encodes the regulatory subunit R1α of PKA, which controls the activation of the catalytic subunit in response to cAMP. Mutations in *PRKAR1A* act as dominant-negative alleles, impairing the release of catalytic subunits and thereby blunting PTH/PTHrP-mediated PKA activation in growth plate chondrocytes [69,70]. Functional studies in knock-in mouse models and patient-derived cells have demonstrated that this signaling blockade leads to abnormal chondrocyte maturation, premature growth plate closure, and profound chondrodysplasia [71]. These mechanistic differences account for the more severe skeletal pathology observed in acrodysostosis compared to pseudohypoparathyroidism.

Primary hyperparathyroidism is exceedingly rare in children, with reported incidence rates ranging from approximately 0.5 to 5 cases per 100,000 person-years [72]. We also identified isolated and syndromic cases in our cohort. *CASR* mutations can be gain-of-function, causing autosomal dominant hypocalcemia type 1, or loss-of-function, leading to familial hypocalciuric hypercalcemia. The recently reported Taiwanese case of *CASR* p.Ile554Asn mutation, supported by in vitro functional assays, further confirms its pathogenic role in FHH. Functional studies demonstrated that this variant destabilizes CASR protein, reduces calcium binding affinity, and blunts downstream MAPK signaling, defects that could be rescued by calcimimetics such as cinacalcet [22]. This highlights the importance of coupling genetic findings with functional characterization, as it not only establishes pathogenicity but also guides potential targeted therapy in patients with FHH or related CASR disorders.

Williams syndrome is a multisystem disorder caused by a 7q11.23 microdeletion involving approximately 26 genes. About 5–15% of infants develop transient hypercalcemia, typically between 12 and 18 months, which usually resolves by early childhood but can occasionally cause nephrocalcinosis or kidney injury [73,74,75]. The mechanism has not been fully understood. PTH levels are usually suppressed, indicating a mechanism distinct from classical hyperparathyroidism, likely involving increased intestinal calcium absorption driven by dysregulated 1,25-dihydroxyvitamin D activity. Haploinsufficiency of genes contributes to abnormal vitamin D sensitivity and calcium regulation [74,76,77,78]. While most children outgrow this phase, subtle disturbances may persist and require long-term surveillance.

Studies suggest that haploinsufficiency of the *WSTF* gene within the 7q11.23 deletion disrupts vitamin D receptor–mediated regulation of *CYP24A1*, reducing the catabolism of active vitamin D metabolites and thereby enhancing the effective activity of vitamin D [79,80]. This mechanism results in increased intestinal calcium absorption and parallels idiopathic infantile hypercalcemia caused by *CYP24A1* mutations [73,81]. In contrast, vitamin D-dependent rickets type 1 arises from *CYP27B1* loss-of-function mutations that abolish 1α-hydroxylase activity, leading to deficient 1,25-dihydroxyvitamin D3 production, hypocalcemia, secondary hyperparathyroidism, and rickets [8,82]. Therefore, WS deletion enhances vitamin D action through impaired degradation, whereas *CYP27B1* mutations impair activation, producing opposite biochemical and clinical outcomes.

### 4.1. Utility of Molecular Testing

This study reinforces the importance of comprehensive molecular testing in pediatric disorders of calcium metabolism. Stepwise testing using MLPA/array CGH, Sanger sequencing, and WES yielded a high diagnostic accuracy. In our study, among the pediatric group, calcium metabolism disorders were most frequently attributed to DiGeorge syndrome. Therefore, when approaching pediatric patients who present with hypocalcemia, clinicians should be alert for associated systemic findings such as T-cell immunodeficiency, thymic aplasia, facial dysmorphism, and congenital heart defects. If these features are present, early genetic testing with MLPA and array CGH should be prioritized to detect potential copy number variations such as 22q11.2 or 10p14 deletions. Additionally, we reported one case of atypical Kabuki syndrome and one case of Williams syndrome. The remaining patients were diagnosed through WES, which identified pathogenic variants in *CASR*, *PRKAR1A*, *GNAS*, and *CYP27B1*. By presenting these serial cases, our findings emphasize that a substantial proportion of calcium metabolism disorders in pediatric patients have a genetic etiology.

### 4.2. Limitations and Future Directions

The study is limited by its single-center, retrospective design, small sample size, and variable follow-up duration, which may result in an underestimate of long-term complications and the prevalence of transient versus permanent disease. Functional studies to validate novel variants were not performed. Future multicenter, prospective studies and functional genomic investigations are needed to clarify the pathogenic mechanisms of novel variants and to optimize personalized management strategies.

## 5. Conclusions

Our study highlights the clinical and genetic heterogeneity of pediatric calcium and phosphate metabolic disorders and emphasizes the pivotal role of molecular testing in diagnosis, management, and genetic counseling. Identification of novel and population-specific variants enriches the global mutational landscape and informs future precision medicine approaches in pediatric endocrinology.

## Figures and Tables

**Table 1:** Patient characteristics.

Patient No.	Age at Clinical Diagnosis	Gender	Clinical Presentation	Ca (mg/dL)	P (mg/dL)	iPTH (pg/mL)	Clinical Diagnosis	Molecular Diagnosis	Mutation Type	Treatment/ Complication	Follow-Up Duration (yrs)
P1	1M	Male	Seizure, hypomagnesemia Brain echo: a few linear calcification over lateral part of basal ganglia on brain echo	5.3	10.2	8.89	Isolated hypoparathyroidism	Idiopathic	-	Calcium carbonate Calcitriol/ Renal echo (-) No hypercalciuria	13
P2	16 Y	Male	Seizure Brain MRI: Calcifications in the bilateral basal ganglia, bilateral subcortical white matter, bilateral thalami, bilateral dentate nuclei and bilateral cerebellum	6.5	8.0	11.6	Isolated hypoparathyroidism	ADH1 CASR, c.2506G>T, p.Val836Leu Heterozygous, AD	Missense	Calcium carbonate Calcitriol/ Hypercalciuria (Urine Ca: 24.2 mg/dL, calcium/CREA ratio 0.32)	1
P3	1 M	Male	Seizure Bilateral relative small kidney	6.4	9.1	7.9	Isolated hypoparathyroidism	ADH1 CASR, c.2506G>A, p.Val836Ile Heterozygous, AD/AR Novel *	Missense	Calcitriol/ No hypercalciuria	4
P4	1 M	Male	Seizure, hypomagnesemia T cell- immunodeficiency, Thymus gland aplasia, Developmental delay PFO (closed)	7.1	9.0	13.4	Type 1 DiGeorge syndrome	22q11 deletion	Micro- deletion	Calcitriol/ No complication	7
P5	25 D	Male	Seizure, T cell- immunodeficiency, Thymus gland aplasia, Facial dysmorphism (micrognathia, low-set ears, hypertelorism), Right side undescended testicle	6.0	9.3	20	Type 1 DiGeorge syndrome	22q11 LCR22-A deletion	Micro- deletion	Calcium carbonate Calcitriol/ No complication	0.5
P6	4 Y	Female	Seizure, Cleft palate and lip, Thymus gland aplasia, VSD (perimembraneous type), PDA, Hypothyroidism, Developmental delay, Left renal dysplastic change with atrophy with mild VUR	7.6	6.7	12.9	Type 2 DiGeorge syndrome	10p.14 deletion	Micro-deletion	Calcitriol Thyroxin/ No complication	8
P7	3 Y	Male	Seizure, Short stature, Hirsutism, Hearing impairment, Bilateral renal parenchymal disease with decreased renal size, Developmental delay, Growth hormone deficiency	6.9	4.5	9.7	Kabuki syndrome	KMT2D, c.5993 A>G, p.Tyr1998Cys, Heterozygous, AD	Missense	Calcitriol	7
P8	9 Y	Female	Short stature, Brachydactyly, Delay puberty, menorrhagia, Psoriasis, chronic arthritis, Osteoporosis with fracture, Hypothyroidism, Cone-shaped epiphyses of fingers	8.3	5.4	177.7	Acrodysostosis	PRKAR1A, c.1004 G>T, p.Arg335Leu Heterozygous, AD	Missense	Calcium carbonate Calcitriol Thyroxin/ No complication	20
P9	14 Y	Female	Short stature, Obesity, Brachydactyly, Irregular menstruation, Hypothyroidism	8.5	5.5	247.4	Pseudohypoparathyroidism	GNAS, c.719-30A>T Heterozygous, AD Novel #	Slice site mutation	Calcium carbonate Calcitriol Thyroxin/ No complication	15
P10	6 Y	Female	Short stature, Brachydactyly, Developmental delay, Hypothyroidism, Growth hormone deficiency, Hypogonadism with primary amenorrhea, Hyperlipidemia	8.1	6.8	330	Pseudohypoparathyroidism	GNAS, c.74dup, p.Ile26AspfrTer28, Heterozygous, AD	Frameshift	Calcitriol Thyroxin/ No complication	15
P11	1 Y	Male	Rickets, Short stature, Genu varum	7.3	2.4	321.7	Vitamin D dependent rickets, type 1	CYP27B1, c.1166 G>A, p.Arg289His c.1319_1325dup, p.Phe443ProfsTer24 Heterozygous, AR	1. Missense 2. Frameshift mutations	Calcium carbonate Calcitriol/ Hypercalciuria (Urine Ca: 37.5 mg/dL, calcium/CREA ratio 0.21) Renal echo suspect bilateral renal parenchymal diseases	15
P12	7 Y	Male	Arthralgia	11.7	3.9	76.2	Primary hyperparathyroidism	FHH CASR, c.1661T>A, p.Ile554ASN Heterozygous, AD	Missense	No medication Follow up	1
P13	2 Y	Male	Failure to thrive, Neurodevelopmental delay, Hypotonia, Hypospadias, Dysmorphism (frontal bossing, hypertelorism, irregularly spaced teeth), Hearing impairment	16.0	3.9	<1.2	Williams syndrome	7q11.23 deletion	Micro-deletion	No medication Diet control Follow up	0.5

* variant was absent from the gnomAD exome and genome datasets (mean coverage: 98.9×; 99.59% of samples with >20× coverage). # variant was absent from the gnomAD exome and genome datasets (mean coverage: 54.9×; 99.79% of samples with >20× coverage). ADH1: Autosomal dominant hypocalcemia type 1, AD: Autosomal dominant, AR: Autosomal recessive, FHH: Familial hypocalciuric hypercalcemia, VSD: Ventricular septal defect, PDA: Patent ductus arteriosus, VUR: Vesicoureteral reflux, PFO: patent foramen ovale.

## Data Availability

The original contributions presented in this study are included in the article material. Further inquiries can be directed to the corresponding authors.
